# State-of-the-Art Advances and Current Applications of Gel-Based Membranes

**DOI:** 10.3390/gels10010039

**Published:** 2024-01-01

**Authors:** Camelia Ungureanu, Silviu Răileanu, Roxana Zgârian, Grațiela Tihan, Cristian Burnei

**Affiliations:** 1 Department of General Chemistry, Faculty of Chemical Engineering and Biotechnologies, The National University of Science and Technology POLITEHNICA Bucharest, Gheorghe Polizu 1-7 Street, 011061 Bucharest, Romania; 2 Department of Automation and Industrial Informatics, Faculty of Automatic Control and Computer Science, The National University of Science and Technology POLITEHNICA Bucharest, Splaiul Independenţei 313 Street, 060042 Bucharest, Romania; silviu.raileanu@upb.ro; 3 Clinical Department of Orthopedics and Traumatology II, Clinical Emergency Hospital, Calea Floreasca 8, 014461 Bucharest, Romania; cristian.burnei@umfcd.ro

**Keywords:** gel, membrane, biomedical applications, water treatment, energy storage

## Abstract

Gel-based membranes, a fusion of polymer networks and liquid components, have emerged as versatile tools in a variety of technological domains thanks to their unique structural and functional attributes. Historically rooted in basic filtration tasks, recent advancements in synthetic strategies have increased the mechanical strength, selectivity, and longevity of these membranes. This review summarizes their evolution, emphasizing breakthroughs that have positioned them at the forefront of cutting-edge applications. They have the potential for desalination and pollutant removal in water treatment processes, delivering efficiency that often surpasses conventional counterparts. The biomedical field has embraced them for drug delivery and tissue engineering, capitalizing on their biocompatibility and tunable properties. Additionally, their pivotal role in energy storage as gel electrolytes in batteries and fuel cells underscores their adaptability. However, despite monumental progress in gel-based membrane research, challenges persist, particularly in scalability and long-term stability. This synthesis provides an overview of the state-of-the-art applications of gel-based membranes and discusses potential strategies to overcome current limitations, laying the foundation for future innovations in this dynamic field.

## 1. Introduction

The concept of membranes can be traced back to ancient civilizations, where simple forms of filtration using natural materials like sand, gravel, and charcoal were employed to purify water [1]. The 19th century saw the birth of dialysis; in 1861, Thomas Graham described the process of separating dissolved substances using “parchment or some analogous membrane” [2]. In the early 20th century, researchers began to use cellulose-based materials to make membranes. In the 1960s, the development of asymmetric cellulose acetate membranes led to the commercialization of the reverse osmosis process, a significant breakthrough for desalination and water purification. Around the same time, ultrafiltration membranes were developed using new polymer materials, enhancing their ability to separate macromolecules. In the latter half of the 20th century, there was significant interest in gas separation [3]. Membranes became a focus for separating gases such as oxygen and nitrogen from air. With the advancement of nanotechnology, new materials like zeolites, metal-organic frameworks (MOFs), and carbon-based materials (e.g., graphene oxide) started being explored for membrane fabrication. Membranes began to be designed not just for separation but also for additional functions such as catalysis, sensing, and antimicrobial activity. Given the increasing concerns over environmental pollution, there was a rising interest in membranes for wastewater treatment, desalination, and other sustainable applications [4,5].

The exploration of membranes for separation processes began with traditional materials like ceramics and polymers. As technology advanced, researchers sought materials that offered more flexibility, adaptability, and specificity. The development of hydrogels, comprising polymer chains crosslinked in a way to retain significant amounts of water, provided a foundation for a new type of membrane. When these hydrogels were further engineered to have controlled pore sizes and mechanical stability, they emerged as viable membrane materials [6,7,8].

The main advantages of the gel-based membranes are their adaptability to different environments due to their tunable nature, better selectivity due to controlled pore sizes, enhanced flux because of their higher water content, and improved biocompatibility, which makes them ideal for biological and medical applications [9,10]. As the search for efficient, adaptable, and sustainable materials in separation technologies intensifies, gel-based membranes have emerged as a beacon of innovation, bridging the gap between functionality and environmental responsibility. The importance of gel-based membranes can be found in multiple areas, such as biomedical applications, water treatment, environmental remediations and energy, durability and stability, sensing and detection, and economic and environmental impact [11,12].

**Figure 1:**
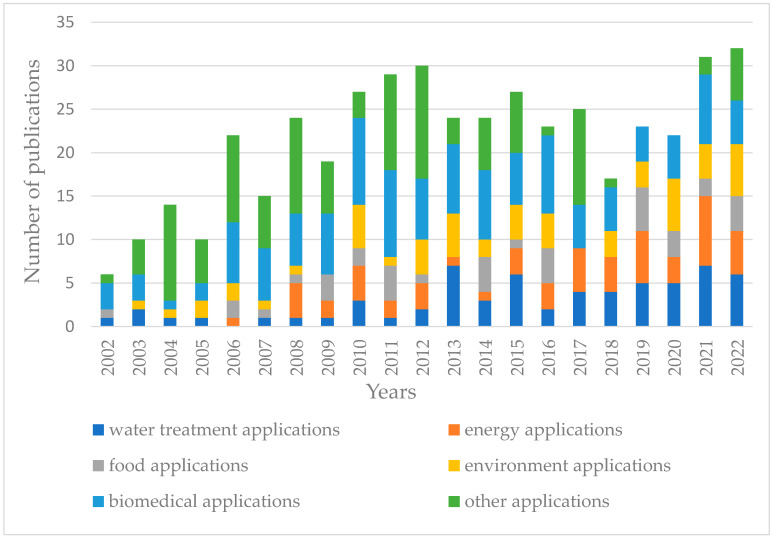
Publication trend (2000–2022) for the applications of the “gel-based membranes” field. (Source of raw data: Document Search - SciELO Citation Index (webofscience.com), accessed on 15 October; search keywords: “gel-based membranes applications”).

Figure 1 shows the publication trend (2000–2022) for the applications of the “gel-based membranes” field. There are multiple applications of gel-based membranes, spanning various fields like water treatment, food application, biomedical application, and energy and environment applications. Their high water content and tunable pore sizes make them especially useful for water purification, including desalination and contaminant removal [13]. Many gel-based membranes are constructed from biopolymers or are designed to be biocompatible, making them particularly suitable for biomedical applications like drug delivery, tissue engineering [14], and hemodialysis [10]. Gel-based membranes made from biopolymers are increasingly being recognized for their potential in wound healing applications. Being derived from natural sources, biopolymers are typically biocompatible, meaning they are less likely to cause adverse reactions when in contact with body tissues [15,16,17,18].

Their ability to house functional groups and change properties in response to specific stimuli also made them suitable for sensing applications [19,20]. In the energy sector, gel-based electrolytes became pivotal in batteries and fuel cells. Their advantages regarding adaptability and ionic conductivity compared to solid electrolytes were significant [21,22]. Their selectivity and adaptability made them good candidates for air purification, carbon capture, and hazardous waste containment [23,24]. In addition to having the flexibility of gels, these membranes were enhanced to maintain their mechanical stability, making them reliable for their long-term use [25]. Due to their efficient separation and low energy requirements, gel-based membranes have the potential to offer sustainable solutions with reduced environmental footprints [26].

This review paper presents a state-of-the-art analysis of advances and current applications of gel-based membranes by highlighting the latest developments and bridging the gaps in existing research by introducing a novel perspective and providing a comprehensive multidisciplinary approach. It not only synthesizes the current body of knowledge but also paves the way for future innovations in different fields (e.g., medical, environmental, and industrial).

## 2. Fundamental Properties of Gel-Based Membranes

Understanding the composition and structure of gel-based membranes is crucial as these factors directly influence their performance in various applications. The versatility in design and adaptability of these membranes comes from the ability to tweak these fundamental properties, which can lead to their widespread utilization in diverse technological fields [9]. Most gel-based membranes are made from a polymeric backbone. This could be natural polymers like agarose, chitosan, and alginate, or synthetic polymers like polyvinyl alcohol (PVA), polyacrylamide (PAM), and polyethylene glycol (PEG). To provide stability and determine the mechanical properties of the gel, crosslinking agents are added. Examples include glutaraldehyde for PVA or N,N’-methylenebisacrylamide for PAM. Depending on the intended use, specific functional monomers can be added to the gel composition, providing specialized properties like increased hydrophilicity, ionic conductivity, or reactive sites for further modifications [27,28,29]. To tailor specific properties or enhance functionality, various additives can be integrated, such as nanoparticles, salts, or organic solvents. At the microscopic level, gel-based membranes exhibit a porous network structure [30,31]. The size and distribution of these pores are crucial determinants of the membrane’s selectivity and permeability. Due to the high water content, these membranes usually exist in a swollen state, where water molecules are trapped within the polymeric matrix. This makes them highly hydrophilic, which in turn affects their permeability and rejection characteristics. The density and nature of the cross-linking play pivotal roles in determining the mechanical strength and flexibility of the membrane [32,33]. A higher cross-linking density usually leads to a stiffer but less swellable membrane. Advanced gel-based membranes might have multiple layers or a composite structure, each layer offering a specific function. For instance, a composite membrane might have a highly selective skin layer and a supportive and mechanically robust sublayer. Gel-based membranes have distinct characteristics that set them apart from other conventional membrane types [34,35]. Due to their gelatinous nature, these membranes exhibit a high degree of swelling, allowing them to retain significant amounts of water. This feature not only enhances their hydrophilicity but also improves their permeation characteristics, especially in aqueous environments [36]. The properties of gel-based membranes, such as pore size, mechanical strength, and chemical functionality, can be easily tailored during the synthesis process by varying the type of monomer, cross-linking agent, and polymerization conditions [37,38]. Some gel-based membranes have the unique ability to heal themselves after being damaged, owing to their dynamic cross-linked network [39,40]. Also, gel-based membranes can be engineered to respond to external stimuli such as pH, temperature, or ionic strength. This responsive behavior can be leveraged for controlled substance release or selective separation applications [41,42]. Unlike rigid ceramic membranes or certain polymeric membranes, gel-based membranes are inherently soft and flexible, which can reduce the risk of fracturing under mechanical stress [43,44]. Despite their soft nature, many gel-based membranes exhibit remarkable stability under various thermal and chemical conditions, especially when designed with specific cross-linkers or additives [45,46]. Also, the high water content and hydrophilic nature of gel-based membranes can reduce the affinity of foulants to the membrane surface, leading to decreased fouling tendencies in applications like water purification [47,48]. Some gel-based membranes, especially those integrated with specific nanoparticles or dyes, can showcase tunable optical properties, which are useful in sensing and detection applications [49]. These distinct properties make gel-based membranes versatile and adaptable to a plethora of applications. Their ability to combine the strengths of both polymeric and ceramic membranes, along with their unique features, underpins their growing significance in the field of advanced separation and filtration technologies.

## 3. Advances in Gel-Based Membrane Technology

**Figure 2:**
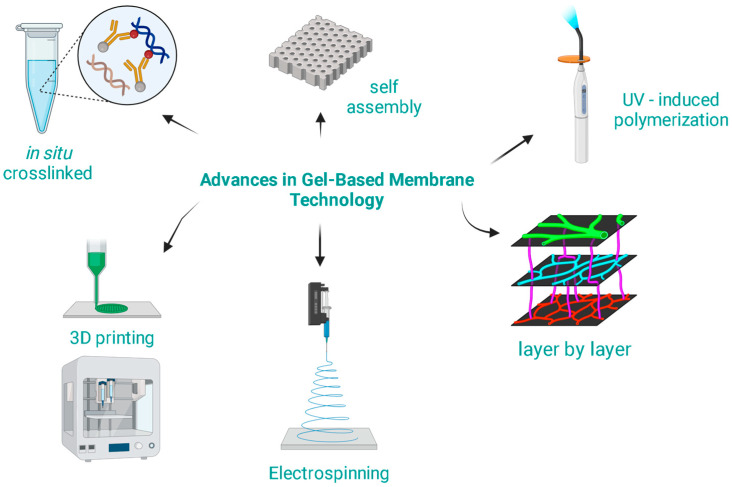
Advances in gel-based membrane technology.

The modern synthesis techniques are not only more efficient but also allow for greater tunability and specificity of the membrane properties [50,51,52]. Some key advances in the synthesis methods for gel-based membranes are presented in Figure 2.

(a) Interfacial polymerization involves the reaction of two immiscible solutions at their interface, leading to the formation of a thin, dense polymeric layer [47]. By adjusting the monomers and conditions, one can tailor the properties of the resulting gel-based membrane. Yuan et al. [48] introduced a novel method for creating thin film composite nanofiltration membranes to tackle global water shortages exacerbated by population growth and water pollution. This technique utilized hydrogel-assisted interfacial polymerization, which incorporated piperazine monomers within a hydrogel that acted as the aqueous phase. This approach facilitated a more uniform polymerization process at the interface, slowed the diffusion of piperazine monomers, and enhanced the mechanical robustness of the resulting polyamide selective layer. Furthermore, the underlying principles contributing to the high permeance were investigated through theoretical simulations of the hydrogel-assisted interfacial polymerization. Significantly, the production method for these hydrogel-thin film composite membranes was straightforward, suggesting potential for cost-effective and scalable manufacturing, which is crucial for widespread application in addressing water scarcity challenges [53,54].
(b) Electrospinning allows for the creation of membranes with high porosity and adjustable fiber diameters. By applying a high voltage to a polymer solution, fibers can be drawn and deposited on a substrate, resulting in nanofibrous gel-based membranes [55,56]. Al-Baadani et al. [55] explored polycaprolactone/gelatin (PCL/Gel) composite membranes, assessing their biocompatibility and controlled drug release capabilities for the first time. Gelatin enhanced osteoblast adhesion and differentiation, while polycaprolactone improved mechanical durability. The study found that adjusting the amount and size of PCL fibers can control the degradation of gelatin and the release patterns of hydrophilic drugs or proteins. Lower PCL content led to a quick dissolution of gelatin fibers and a rapid drug release within one week. Conversely, higher PCL levels slowed the release rate, extending the duration to over two weeks, which could be beneficial for bone regeneration applications. The findings indicate that PCL/Gel composite membranes fabricated via co-electrospinning could be an effective method for creating customizable drug delivery systems that support bone healing.
(c) Layer-by-Layer (LbL) assembly uses the alternate deposition of positively and negatively charged polymers, building up multi-layered structures. The method allows for the precise control of membrane thickness and functionality. LbL techniques can help in crafting multilayered membranes, where each layer can cater to a specific separation requirement, offering improved overall selectivity. In a review paper, Liu et al. [57] focused on multi-layered hydrogels, which are preferred materials for biomedical applications due to their organized functional layers. It compiled recent advancements in multi-layered hydrogels, categorizing them based on their fabrication techniques such as layer-by-layer self-assembly, stepwise, photo-polymerization, and sequential electrospinning. The review also examined the morphology of these hydrogels and their various biomedical uses. It concluded by addressing the current challenges faced in the development of multi-layered hydrogels and suggested that 3D printing technology may offer innovative solutions for designing these materials, potentially broadening their biomedical applications.
(d) During in situ cross-linking, the membrane is formed by introducing cross-linking agents directly into the polymer solution or gel, triggering cross-linking in place. This can provide enhanced mechanical stability and tailor the swelling behavior of the gel-based membrane [58,59]. Leone et al. [53] studied the production of such hydrogels designed for wound healing, particularly in treating complex blast injuries. In situ-forming hydrogels are emerging as versatile biomaterials for patient-specific biomedical applications like cell therapy and drug delivery. Researchers developed bionanocomposite hydrogels by integrating oxidized polysaccharides (which have aldehyde groups), chitosan (rich in amine groups), and nanostructured zinc oxide. They thoroughly examined the physicochemical properties of these components, their cytotoxicity towards HaCat skin cells, and the release profile of zinc ions on synthetic skin models. The hydrogels formed rapidly in situ, exhibited no toxicity at functional levels for HaCat cells, and successfully released Zn^2+^ ions, indicating the potential to promote wound healing.
(e) UV-induced polymerization uses ultraviolet light to initiate polymerization and cross-linking within the gel precursor. It offers rapid synthesis and the potential for spatial control over membrane properties [60,61]. Siccardi et al. [55] introduced a novel approach using UV-induced, solvent-free radical copolymerization to create a solid polymer electrolyte with enhanced properties. Solid polymer electrolytes offer a safer alternative for lithium metal batteries, but their practical use is hindered by low ionic conductivity and poor cyclability at room temperature. When activated with a small amount of liquid electrolyte, these polymers demonstrated high thermal resistance, good lithium-ion conductivity, and a wide electrochemical window. The polymers exhibited excellent interfacial stability, enabling stable lithium metal plating and stripping at room temperature. This research led to a significant advancement toward developing safer, room-temperature operable, self-healing quasi-solid-state lithium metal batteries.
(f) With the self-assembly method, some gel-based systems can spontaneously organize into structured networks through non-covalent interactions like hydrogen bonding, electrostatic forces, or π-π stacking. This bottom-up approach can lead to membranes with highly ordered nanostructures [62,63]. Braun et al. [57] investigated the self-assembly of the hydrogel-forming peptide, revealing the microscopic steps of its formation. By applying theoretical models of linear polymerization to kinetic data, it was discovered that peptide fibril formation is predominantly driven by fibril-catalyzed secondary nucleation. Furthermore, the peptide’s self-assembly processes exhibited enzyme-like saturation, indicating that they are not simply chemical reactions but are regulated in a manner like biological systems. The study quantified the rates of these processes at various concentrations and the evolution of these rates throughout the assembly. This novel mechanistic approach, distinct from traditional material science methods, could lead to more a sophisticated design and application of self-assembling hydrogels in medicine.
(g) Microfluidic fabrication allows for the controlled synthesis of gel particles or fibers with uniform sizes and shapes. When these are used to fabricate membranes, they can offer highly reproducible transport properties [64,65]. Correa et al. [58] explored the biofabrication of stable, aligned collagen hydrogels within microfluidic devices, aiming to improve tissue and organ models for extended culture times. Collagen-alginate microgels were created by cross-linking with calcium ions within a microfluidic channel, using a chitosan membrane to allow ion diffusion without convection. These gels formed rapidly into isolated structures and their growth was self-regulating. By adjusting the calcium concentration and the flow rate of the collagen-alginate solution, gel thickness could be precisely controlled between 30 and 200 μm. Less calcium and higher flow rates resulted in more compressed gels, especially further from the pores. The study also showed that the gels allowed size-dependent diffusion of molecules, making them suitable for on-chip models that require the invasion of a dense extracellular matrix, cancer growth, and targeted drug delivery. This demonstrates the potential of controlled physicochemical parameters for collagen gel formation in microfluidic applications.
(h) Incorporation of nanomaterials: Modern synthesis methods have adapted to integrate various nanomaterials like metal nanoparticles, carbon nanotubes, or graphene oxide into the gel matrix. This enhances the functional properties of the membrane, from improved mechanical strength to specialized separation characteristics [66,67].
(i) 3D Printing techniques are being explored to create gel-based membranes with intricate structures, customizable geometries, and multi-functional regions [68,69]. Tayebi et al. [63] highlighted the creation of scaffolds designed for the cultivation of full-thickness oral mucosa, representing a type of heterogeneous tissue. By exploring these dimensions, the paper provided insights into the nuanced production of biologically relevant models using 3D printing technology, which holds potential for advancing tissue engineering and regenerative medicine. Biological membranes, while seemingly two-dimensional, possess intricate structures extending into the third dimension. Three-dimensional printing, particularly through layer-by-layer assembly, emerged as a sophisticated technique for crafting models that embody this complexity. Nonetheless, printing certain hydrogels like gelatin can be challenging due to their unique rheological properties. The authors tackled these challenges by analyzing the complexities of 3D printing gelatin, proposing a reproducible method to surmount the associated experimental hurdles, and detailing the design specifications and fabrication process for 3D printed gelatin membranes.

These modern synthesis methods underscore the versatility and adaptability of gel-based membrane technology. By leveraging these techniques, researchers can design membranes for specific applications with unprecedented precision, paving the way for innovations across diverse industrial domains.

The advancements in gel-based membrane technology have ushered in significant enhancements in mechanical strength, selectivity, and longevity. The density and nature of cross-linking have been optimized to provide higher mechanical robustness [70]. For instance, dual cross-linking, involving both physical and chemical bonds, can significantly boost strength [71]. The introduction of nanofillers like silica nanoparticles, carbon nanotubes, or graphene oxide can reinforce the gel matrix, leading to improved tensile strength and toughness [72]. Creating hybrid structures by incorporating both organic and inorganic components can marry the flexibility of polymers with the rigidity of inorganic materials, leading to enhanced mechanical properties [73]. Advanced synthesis methods allow for precise control over pore sizes, enabling high selectivity based on the size of molecules or ions. The introduction of specific functional groups or ligands can enhance selectivity based on chemical interactions, such as hydrogen bonding, electrostatic attractions, or even affinity-based separations [74]. Incorporation of hydrophilic groups or zwitterionic components can provide antifouling properties, reducing membrane fouling and thereby extending its lifespan for applications. Chemical modifications or the inclusion of stabilizing agents can render the membranes more resistant to harsh conditions like extreme pH, high temperatures, or aggressive solvents [75]. Some modern gel-based membranes are imbued with self-healing capabilities, where minor damage can be auto-repaired, thus prolonging their operational lifespan [76,77]. The addition of protective coatings or layers can shield the membrane from mechanical abrasions, aggressive chemicals, or microbial attacks, thereby extending its durability [78]. The development of efficient cleaning-in-place (CIP) and maintenance protocols has further bolstered the longevity of gel-based membranes in industrial applications [79].

These enhancements in mechanical strength, selectivity, and longevity underline the commitment of researchers and industries to optimize gel-based membrane technology. Such improvements ensure that these membranes can meet the rigorous demands of contemporary applications while maintaining operational efficiency over extended periods. Incorporating nanoparticles or functional groups into membranes has provided an avenue to tailor membrane properties to specific needs [80]. This customizable approach ensures that the resultant membranes are not only more efficient but also versatile in handling a wide range of applications. For example, by incorporating metal and metal oxide nanoparticles like silver (Ag), gold (Au), titanium dioxide (TiO_2_), and zinc oxide (ZnO), they can impart antimicrobial properties, improve thermal stability, and enhance mechanical strength [81,82,83]. Also, incorporating magnetic nanoparticles is useful in applications where remote actuation or controlled movement is required, like in drug delivery or targeted separations. Nanoparticles can add size-exclusion properties or even specific affinity interactions that can enhance the selectivity of membranes [84]. Certain nanoparticles can improve the porosity and hydrophilicity of the membranes, leading to improved permeation rates [85]. Incorporating groups like -OH, -COOH, or -NH_2_ can also enhance the hydrophilicity of the membrane, reducing fouling and improving water flux. Zwitterionic Groups have both positive and negative charges, which can greatly reduce protein or microbial fouling due to their unique surface properties [86].

Functional groups like epoxy, carboxyl, or amine can allow for further modifications, tethering of other molecules, or even specific interactions with target substances [87].

The introduction of charged moieties (like sulfonic or quaternary ammonium groups) can enhance the ionic selectivity of the membrane, useful in processes like desalination or ion exchange [88]. Functional groups that have a specific affinity for certain contaminants (e.g., chelating agents for heavy metal capture) can be introduced into membranes to provide selectivity based on chemical interactions [89].

The integration of nanoparticles and functional groups into water treatment membranes has set a new benchmark for efficiency, selectivity, and sustainability. These advanced membranes not only ensure cleaner water but also promise more energy-efficient and eco-friendly water treatment solutions. Gel-based membranes, with their unique properties and adaptability, hold great potential in revolutionizing water treatment processes, providing both efficiency and sustainability [90].

## 4. Applications of Gel-Based Membranes

**Figure 3:**
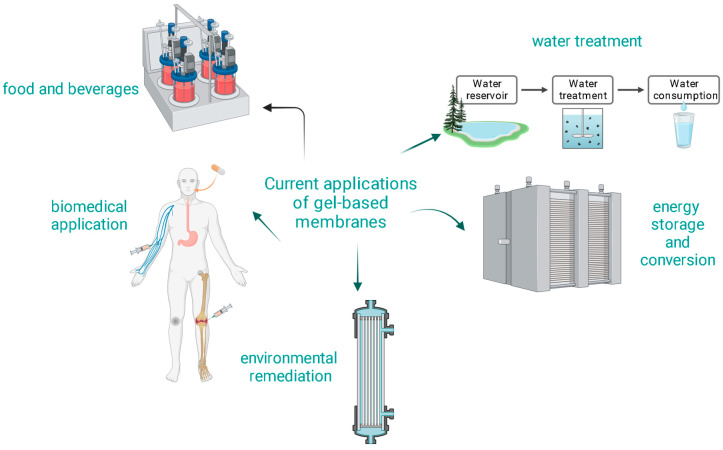
Current applications of gel-based membranes.

One of the defining characteristics of gel-based membranes is their unique combination of elasticity and mechanical strength. This combination allows for potential self-healing properties and resistance to physical damage [91]. The hydrophilic and smooth nature of gel-based membranes can lead to reduced biofouling and organic fouling, enhancing the membrane’s lifespan [11]. The porous structure of gel-based membranes can be finely tuned to achieve the desired permeability and selectivity [92]. Gel-based membranes, especially those derived from natural polymers, are often biocompatible, making them suitable for applications where avoiding toxic by-products is essential [93]. Figure 3 shows some current applications of gel-based membranes.

### 4.1. Applications in Water Treatment

By embedding nanoparticles or other agents, gel-based membranes can be endowed with multiple functionalities, enabling them to perform several tasks simultaneously, like separation and catalysis [94]. 

Gel-based membranes for water treatment combine the advantages of hydrophilicity, selectivity, and anti-fouling properties, making them highly effective for various water purification needs [11,95]. Gel-based membranes are typically synthesized from hydrophilic polymers. Common choices include polyvinyl alcohol (PVA), polyethylene glycol (PEG), and various polysaccharides. To form a stable gel structure, these polymers are often cross-linked. Chemical cross-linkers like glutaraldehyde or physical methods such as UV irradiation can be used. Their structure allows for the selective permeation of water molecules while retaining contaminants like heavy metals, organic pollutants, or pathogens. The hydrophilic nature helps resist fouling, a common challenge in membrane-based water treatment. These membranes must be chemically stable in various water conditions, including different pH levels and the presence of various contaminants. While gel membranes are flexible, they need sufficient mechanical strength to withstand operational pressures in water treatment systems. Their design and synthesis are tailored to the specific requirements of the water treatment application, such as desalination (removing salts to produce potable water from seawater or brackish water), pollutant removal (effective in trapping heavy metals like lead, arsenic, and mercury from industrial wastewater), removal of organic compounds (e.g., endocrine-disrupting chemicals, pharmaceuticals, and personal care products), pathogen filtration (with the possibility to be tailored for the removal of bacteria, viruses, and other pathogens), and reduction of hardness-causing ions like calcium and magnesium [96,97,98,99,100,101].

Table 1 presents some applications of gel-based membranes in water treatment.

### 4.2. Biomedical Applications of Gel-Based Membranes

The adaptability and intrinsic properties of gel-based membranes make them immensely suitable for a range of biomedical applications. Gel-based membranes made from biopolymers are increasingly being recognized for their potential in wound healing applications. Examples of biopolymers used in these membranes include collagen, chitosan, alginate, and hyaluronic acid, among others [102]. Each of these has unique properties, making them suitable for different wound healing applications, from minor cuts and abrasions to more severe burns and surgical wounds. These membranes offer several advantages like biocompatibility, moisture maintenance, permeability, natural degradation, drug delivery, and structural and mechanical properties [103,104]. Biopolymers are often biodegradable, meaning they can break down naturally in the body, eliminating the need for removal and reducing the risk of chronic inflammation [105]. Gel-based biopolymer membranes can maintain a moist environment, which is beneficial for wound healing [106]. A moist environment can facilitate cell migration and proliferation, essential for tissue regeneration. These membranes are often semi-permeable, allowing for gas exchange while keeping out pathogens. This characteristic is crucial for protecting the wound from infections while allowing it to “breathe” [107]. Some biopolymer membranes can be engineered to deliver therapeutic agents directly to the wound site, such as antibiotics, pain relievers, or growth factors to promote healing. Gel-based biopolymer membranes can be tailored to have specific structural and mechanical properties, such as elasticity and strength, to match the needs of different types of wounds [108,109,110]. 

Hemodialysis membranes are typically synthesized from biocompatible hydrogels, such as cellulose or synthetic polymers like polyether sulfone. Cross-linking methods, like radiation or chemical cross-linking, are employed to enhance membrane stability. These membranes have a porous structure with a controlled pore size, allowing the selective diffusion of waste products from the blood while retaining essential proteins and blood cells. Hemodialysis membranes exhibit high biocompatibility, hemocompatibility, and good mechanical strength. They have a high surface area for efficient dialysis, and their properties are optimized to prevent clotting and inflammation during treatment.

Transdermal drug delivery membranes are synthesized from biocompatible and permeable hydrogels. The synthesis process may involve incorporating drugs or active compounds into the gel matrix. These membranes are typically thin and flexible, designed to adhere to the skin’s surface. They control drug release through diffusion or other controlled mechanisms. Transdermal drug delivery membranes provide controlled release of drugs over time, offering a convenient and non-invasive method for drug administration [111,112,113,114,115,116,117,118,119].

Their potential to be engineered precisely as per the application’s demands ensures that they will remain at the forefront of biomedical research and innovations (Table 2).

### 4.3. Applications of Gel-Based Membranes in Energy Storage and Conversion

In the arena of energy storage and conversion (Table 3), gel-based membranes offer a suite of advantages that can potentially lead to more efficient, safer, and more durable devices. As research in this domain continues, it is highly plausible that these membranes will play a significant role in the next generation of energy technologies. The role of gel-based membranes in energy storage and conversion is multifaceted, encompassing electrolytes in batteries and fuel cells, separators in supercapacitors, and functional components in solar cells and electrochromic devices. Their design and synthesis are heavily tailored to meet the specific electrical, ionic, and mechanical demands of each application [120,121].

Proton-conductive gel-based membranes for fuel cells are synthesized from sulfonated or acid-functionalized hydrogels. The synthesis process often includes sulfonation or functionalization of the polymer chains. These membranes have a well-defined structure with proton-conductive groups distributed throughout the polymer matrix. The structure ensures efficient proton transport. Fuel cell membranes exhibit high proton conductivity, chemical stability, and mechanical strength. They enable the efficient conversion of hydrogen and oxygen into electricity. The synthesis often involves conductive polymers like polyaniline, polypyrrole, or polythiophene to ensure electrical conductivity. To enhance their electrochemical properties, electroactive materials such as metal oxides or conductive nanoparticles may be incorporated. For applications like batteries and fuel cells, the ionic conductivity of the membrane is crucial. This is often achieved by incorporating ionic liquids or salts into the gel matrix. Creating composite membranes with materials like graphene, carbon nanotubes, or metal-organic frameworks can enhance electrical and ionic conductivity, as well as mechanical strength.

Gel-based membranes are used in lithium-ion batteries, sodium-ion batteries, and other types of rechargeable batteries, serving as electrolytes that facilitate ion transport [122,123,124,125]. They are used as proton exchange membranes (PEMs) or electrolytes in fuel cells, including PEM fuel cells, facilitating the transport of ions while preventing electron flow [126,127,128,129]. In supercapacitors, they act as separators and electrolytes, contributing to the device’s overall capacitance and energy density [130]. Some gel-based membranes are used in photovoltaic cells, particularly in organic solar cells, as they can be engineered to improve light absorption and charge transport [131,132]. Also, they are used in smart windows and displays, where their ability to conduct ions plays a crucial role in the color-changing mechanism [133].

### 4.4. Applications of Gel-Based Membranes in Food and Beverages

Gel-based membranes have also been used in the food and beverage industry (Table 4). They can act as selective barriers to oxygen, carbon dioxide, and ethylene, which is crucial in food packaging for shelf life extension. In the context of the food and beverage industry, gel-based membranes offer solutions that can enhance product quality, safety, and sensory attributes. Their potential to be customized for specific applications ensures that they will continue to be of interest in food science and technology research. In the food and beverage industry, gel-based membranes offer innovative solutions for packaging, processing, and ensuring product quality and safety. Membranes for food applications are typically made from food-grade, non-toxic materials like alginate, or pectin. Natural extracts or antimicrobial agents can be incorporated to extend shelf life and improve food safety. The synthesis often focuses on achieving specific permeability properties to target certain molecules, such as gases, flavors, or nutrients. Stability under various temperature conditions is important for processing and storage. Used in packaging to extend shelf life, improve safety, or maintain quality. For example, oxygen-scavenging membranes help prevent oxidation in packaged foods. They can be applied as thin, edible coatings on fruits and vegetables to extend shelf life and reduce spoilage [134]. In beverage processing, these membranes can be used for selective infusion or removal of flavors, colors, or nutrients They can also be used in the dairy industry for processes like lactose reduction and protein concentration [135,136,137,138,139,140].

### 4.5. Applications of Some Gel-Based Membranes in Environmental Remediation

Gel-based membranes, due to their versatility and adaptability, have been explored for various environmental remediation applications (Table 5). Due to their multifunctionality and adaptability, they hold great promise in addressing the current pressing environmental challenges. Their ability to be tailored for specific contaminants ensures targeted and effective remediation. As research progresses, these membranes will likely play a more prominent role in sustainable environmental solutions.

In environmental remediation, gel-based membranes offer the advantage of tailored selectivity for various pollutants, high efficiency, and the potential for regeneration and reuse. Their application spans from water treatment, targeting a variety of pollutants, to air purification and greenhouse gas capture, highlighting their versatility and importance in addressing environmental challenges [141,142].

**Table 5:** Applications of some Gel-Based Membranes in Environmental Remediation.

Applications	Characteristics of Gel-Based Membranes	References
**Air Purification**		
Volatile Organic Compound (VOC) Removal	Gel-based membranes can be engineered to selectively capture and remove VOCs from indoor and industrial air streams. Their porous structure allows for high surface area interaction with contaminants.	[143]
Particulate Matter Capture	Certain gel membranes can be designed to effectively trap particulate matter, including fine and ultrafine particles, which are detrimental to human health.	[144]
Bioaerosol Capture	With functionalized surfaces, gel-based membranes can capture and potentially neutralize bioaerosols, including bacteria, viruses, and fungal spores, contributing to cleaner air in healthcare and public spaces.	[145]
**Carbon Capture**		
Enhanced Gas Selectivity	Gel-based membranes, especially when embedded with functional groups or materials that have an affinity for CO_2_, can effectively separate carbon dioxide from gas mixtures, such as flue gases from power plants.	[146]
Reduced Energy Penalty	Traditional carbon capture methods can be energy-intensive. Gel-based membranes, with their potential for high permeability and selectivity, can reduce the energy penalty associated with the separation process.	[147]
Durability	Properly designed gel-based membranes can offer stability and resistance to fouling, ensuring long-term performance in CO_2_ capture applications.	[148]
**Hazardous Waste Containment**		
Heavy Metal Removal	Gel-based membranes, when functionalized with chelating agents or other selective groups, can effectively bind and remove heavy metals from wastewater.	[98]
Organic Pollutant Capture	Membranes can be tailored to selectively adsorb organic contaminants, including oil, pesticides, and certain industrial chemicals, from water sources.	[51]
Radioactive Waste Containment	Some gel-based membranes are being explored for their potential in containing and isolating radioactive compounds, especially in liquid radioactive waste treatment.	[149]
Encapsulation and Immobilization	Hazardous compounds can be effectively encapsulated within gel matrices, preventing their migration and leaching into the environment. This is particularly useful for the long-term containment of certain pollutants.	[150]

The polymers chosen for synthesis, such as polyacrylamide, chitosan, or alginate, often have specific affinities for certain pollutants. The introduction of functional groups or active sites that can selectively bind to or react with specific contaminants is common. This can include groups that target heavy metals, organic pollutants, or radioactive materials. Materials like activated carbon, zeolites, or metal-organic frameworks can be incorporated into the membrane matrix to enhance adsorption properties. The development of composite membranes, which might include inorganic materials or nanoparticles, can improve mechanical strength, chemical resistance, and pollutant removal efficiency. The structure is typically porous, allowing for efficient flow-through of water while trapping contaminants. These membranes often exhibit swelling behavior, which can be tuned for specific applications to enhance pollutant capture. The morphology, including pore size and distribution, is often optimized for specific types of pollutants [143,144,145].

Gel-based membranes are effective in capturing heavy metals from industrial wastewater, such as lead, mercury, and cadmium, and are used to remove organic contaminants, including pesticides, pharmaceuticals, and dyes from water. They can be specialized for the separation of oil from water, which is crucial in treating oil spills and industrial emulsions. Also, some gel-based membranes are designed to capture radioactive isotopes, aiding in the cleanup of nuclear waste. They can also be applied in gas separation processes, removing pollutants like sulfur dioxide or nitrogen oxides from industrial emissions. Specialized gel membranes can be used for carbon capture, helping to reduce greenhouse gas emissions [51,98,146,147,148,149,150].

Table 6 shows important information about the production of the gel-based membrane. 

## 5. Challenges and Future Perspectives of Gel-Based Membranes

While gel-based membranes have shown remarkable potential, addressing the current challenges is crucial for their widespread adoption. The ongoing research and technological innovations in this domain are promising and suggest a bright future for these versatile materials. One of the key challenges with gel-based membranes is their mechanical fragility compared to other membrane types. They may not withstand high pressures or aggressive operating conditions over long durations. Over time, gel-based membranes can face fouling, especially when used in wastewater treatment or other applications with high organic loadings. This reduces their performance and lifespan. Some gel membranes tend to swell excessively in the presence of certain solvents or conditions, leading to a loss in their selective properties. Manufacturing large-scale, uniform, and defect-free gel-based membranes, especially for industrial applications, can be challenging and costly. Introducing cross-links in the polymer network can enhance the mechanical strength and stability of the membranes. Incorporating nanoparticles or nano-fillers into the gel matrix can improve properties such as mechanical strength, selectivity, and resistance to fouling. The use of state-of-the-art manufacturing techniques, like 3D printing, could enable the production of customized and defect-free membranes at larger scales. Adding specific functional groups or coatings can enhance selectivity, reduce fouling, and improve overall performance. Developing efficient cleaning techniques that do not compromise membrane integrity can extend the life and efficiency of these membranes. The integration of sensing elements or responsive components can lead to ‘smart’ membranes that adapt or respond to changes in their environment, improving efficiency and selectivity. With an increasing emphasis on sustainability, there will be a rise in the research and development of biodegradable or compostable gel-based membranes. Combining gel-based membranes with other technologies, such as advanced oxidation processes or biofiltration, can lead to integrated systems with enhanced efficiency. In the biomedical domain, gel-based membranes might be customized for individual needs, particularly in areas like drug delivery or tissue engineering. As the technology matures and overcomes its current limitations, there will likely be an uptick in the commercial applications of gel-based membranes in various industries, from environmental to biomedical fields.

## 6. Conclusions

Gel-based membranes, with their unique blend of structural, chemical, and functional properties, have undeniably carved a niche in modern technological applications. From purifying water to enhancing energy storage, safeguarding the environment, and pushing the boundaries in biomedical applications, they have demonstrated unparalleled versatility. Their tailoring ability allows for bespoke solutions to challenges across various industries, emphasizing their centrality in addressing contemporary issues. However, as with any evolving technology, they come with their set of challenges. Future research must not only aim to harness their potential but also innovatively address their limitations. Advanced research, cross-disciplinary collaborations, and industrial partnerships are pivotal in this regard.

Looking forward, the horizon for gel-based membranes seems promising. As we tread into an era marked by sustainability, efficiency, and precision, these membranes are poised to play a pivotal role. Their continued evolution will not only augment existing technologies but also pave the way for novel applications yet to be envisioned. The future holds a multitude of opportunities for gel-based membrane applications due to their multifaceted potential.

## Figures and Tables

**Table 1:** Applications of Gel-Based Membranes in Water Treatment.

Processes	Characteristics of Gel-Based Membranes	References
**Desalination Processes**		
Reverse Osmosis (RO)	Enhanced flexibility and can be tailored for specific pore sizes, enabling improved salt rejection. Their hydrophilic nature can also enhance water permeability, reducing the energy input required.	[96]
Forward Osmosis (FO)	They have the innate ability to swell, which can be utilized in FO processes. Their hydrophilic nature ensures better water transport, mitigating fouling.	[97]
Electrodialysis (ED)	While not as commonly used as other materials, gel-based membranes with embedded charged functional groups or nanoparticles can potentially be employed in ED for selective ion transport.	
**Removal of Pollutants and Heavy Metals**		
Adsorptive Removal	Some gel-based membranes can be modified or embedded with materials like activated carbon, enhancing their capacity to adsorb organic pollutants.	[98]
Affinity-based Removal	Functional groups with specific binding affinities can be incorporated into gel matrices. For instance, gels with chelating agents can selectively bind and remove heavy metals.	[99]
Photocatalytic Decomposition	By incorporating photocatalytic nanoparticles within gel-based membranes, pollutants can be decomposed under light exposure.	[100]
Magnetic Response	Gel-based membranes infused with magnetic nanoparticles can be used for magnetically guided pollutant capture and easier membrane regeneration.	[101]

**Table 2:** Applications of some Gel-Based Membranes in Biomedical Applications.

Applications	Characteristics of Gel-Based Membranes	References
**Drug Delivery Systems**		
Controlled Release	Gel-based membranes can control the rate at which drugs are released. This is particularly useful for sustained-release formulations where medication needs to be delivered over an extended period.	[111]
Targeted Delivery	With the incorporation of functional groups or nanoparticles, gel-based membranes can be engineered to release drugs in response to specific triggers, such as pH changes, temperature, or even the presence of specific biomolecules.	[112]
Layered Systems	Multi-layered gel membranes can be designed to release multiple drugs in a sequential or concurrent manner, allowing for combination therapies.	[113]
**Tissue Engineering Scaffolds**		
Biocompatibility and Biodegradability	Many gel-based membranes, especially those derived from natural sources, are biocompatible and can be tailored for biodegradability. This ensures that they support cell growth without inducing adverse reactions and can degrade over time to be replaced by natural tissues.	[114]
Pore Architecture	The porosity of gel-based scaffolds can be tailored to mimic the extracellular matrix, providing an optimal environment for cells to proliferate and differentiate.	[115]
Growth Factor Incorporation	Gel-based membranes can be infused with growth factors or other bioactive agents to stimulate tissue regeneration.	[116]
Mechanical Support	While maintaining flexibility, gel-based membranes can provide the necessary mechanical support for developing tissues, especially soft tissues.	
**Hemodialysis**		
Enhanced Selectivity	Gel-based membranes can be designed with precise pore sizes to selectively remove waste products from the blood while retaining essential molecules.	[52]
Reduced Fouling	Their hydrophilic nature can reduce protein adhesion and fouling, which is a common challenge in hemodialysis processes.	[117]
Biocompatibility	Being in direct contact with blood, hemodialysis membranes need to be highly biocompatible to prevent adverse reactions. Gel-based membranes, especially those derived from natural materials, can meet this requirement.	[118]
Functionalization	Gel membranes can be functionalized with specific agents or coatings to further enhance their performance, such as antithrombogenic coatings to prevent blood clotting.	[119]

**Table 3:** Applications of some Gel-Based Membranes in Energy Storage and Conversion.

Applications	Characteristics of Gel-Based Membranes	References
**Gel-based Electrolytes in Batteries**		
Lithium-Ion Batteries (LIBs)	Gel-based electrolytes can enhance the safety profile of LIBs. They can prevent dendrite growth, a major issue with liquid electrolytes, and reduce risks associated with leakage and combustion. The ionic conductivity of these gel electrolytes can be tailored to be competitive with traditional liquid electrolytes.	[122]
Solid-State Batteries	Gel-based membranes can act as a bridge between liquid electrolytes and true solid-state systems, offering flexibility and ionic conductivity without the leakage risks associated with liquids.	[123]
Enhanced Safety and Stability	Gel electrolytes substantially reduce the risk of leakage, which is a common concern with liquid electrolytes. Moreover, they can suppress dendritic lithium growth, which can cause short circuits in batteries.	[124]
Broad Operating Temperatures	Gel-based electrolytes can function over a broader temperature range compared to some liquid counterparts, enhancing battery performance in extreme conditions.	[125]
**Fuel Cells**		
Proton Exchange Membrane Fuel Cells (PEMFCs)	Gel-based membranes can be used as proton exchange membranes in PEMFCs. They can offer good proton conductivity while being less permeable to gases like hydrogen and oxygen, which improves the fuel cell’s efficiency.	[126]
Direct Methanol Fuel Cells (DMFCs)	One challenge with DMFCs is methanol crossover, which lowers the cell’s efficiency. Gel-based membranes can potentially reduce this crossover due to their structure and customizable porosity.	[127]
High Thermal and Mechanical Stability	Fuel cells often operate under conditions where they are exposed to high temperatures and varying pressures. Gel-based membranes can provide the necessary stability under these conditions, ensuring the longevity and efficiency of the fuel cell.	[128]
Tailored Ionic Conductivity	By incorporating specific functional groups or inorganic fillers, the ionic conductivity of gel-based membranes can be enhanced, making them suitable for efficient operation in fuel cells.	[129]

**Table 4:** Applications of Gel-Based Membranes in Food and Beverages.

Application	Characteristics of Gel-Based Membranes	References
**Encapsulation of Flavors and Fragrances**		
Controlled Release	Gel-based membranes can encapsulate flavors or fragrances, allowing for their controlled release. This ensures that the sensory experience (e.g., aroma or taste) is sustained over a longer period or is triggered under specific conditions.	[135]
Protection	Delicate flavors and fragrances that might be sensitive to environmental factors like oxygen, moisture, or light can be protected within gel matrices. This helps in preserving their integrity and enhancing their shelf life.	[136]
Improved Solubility	Certain flavors or fragrances might not be easily soluble in their product matrix. Encapsulation within hydrophilic gel-based membranes can improve their dispersion and solubility in aqueous systems.	[135]
**Removal of Undesirable Compounds from Beverages**		
Selective Separation	Gel-based membranes can be tailored for the selective removal of undesired compounds, such as certain bitter compounds, excess caffeine, or contaminants from beverages without affecting the beneficial compounds or the desired flavor profile.	[137]
Dealcoholization	In the production of non-alcoholic or low-alcohol beers and wines, gel-based membranes can selectively remove ethanol while retaining the essential flavors and aromatic compounds.	[138]
Purification	Beverages, especially fruit juices, might contain suspended particles, pectin, or certain enzymes that can affect their clarity and shelf life. Gel-based membranes can aid in the clarification and stabilization of these beverages.	[139]
Bioactive Enrichment	Gel-based membranes can also be used in concentration processes where the aim is to increase the content of specific bioactive compounds, such as antioxidants in fruit juices, without concentrating other undesired compounds.	[140]

**Table 6:** Some applications of Gel-Based Membranes.

Application	Backbone Polymer	Cross-Linking Agent	Integrated Additives	Synthesis Technique	Selectivity/ Permeability	Ref.
wound skin dressing	PVA, gelatin	-	Zinc (II)–penicillin complex	esterification	-	[151]
drug delivery	PVA, gelatin	-	Salicylic acid (drug)	esterification	-	[152]
membrane chromatography	Natrix: polyacrylate hydrogel with a high density of carboxylate groups;	-	-		-	[153]
Tendon repair	XG/GG/HA	EDC/NHS	-	cross-linking		[154]
Solid-State Batteries	polyacrylamide (PAM)-based alkaline gel electrode	-	-	Solution casting	Specific Capacity 720 mA h/g	[155]
Biological applications	Double network (DN) gels: triblock copolymers, polybutyl methacrylate-bpolymethacrylic acid-b-polybutyl methacrylate + Aam, DMA, and DEA		-	combining noncovalent DN strategy and spin-coating method	-	[156]
Water filtration	hydrogel			in situ graft polymerization		[157]
Sensors	hydrogel			pipette tip	sensitive detection of 0.5 μM–500 mM potassium ions	[158]
Nanofiltration	Kevlar hydrogel		Hydrogel/PA thin layer	interfacial polymerization	ultrahigh high permeances of 52.8 and 62.9 L m^−2^ h^−1^ bar^−1^ while maintaining satisfactorily high rejections of 96.4% and 93.5% for Na_2_SO_4_	[54]
Quasi-solid state sodium ion battery	PVDF-HFP and PBMA			Solution casting	ionic conductivity of 1.086 × 10^−3^ S cm^−1^	[159]
Adsorptive features for Pb^2+^, UO_2_^2+^, and Th^4+^	polyacrylamide hydrogel		chitosan	polymerization	-	[160]
Encapsulation of pectinase	polyacrylamide gel	-	-	polymerization	-	[161]

## Data Availability

Not applicable.

## Abbreviations

MOFs	metal-organic frameworks
PVA	polyvinyl alcohol
PAM	polyacrylamide
PEG	polyethylene glycol
LbL	Layer-by-Layer
CIP	cleaning-in-place
Ag	silver
Au	gold
TiO_2_	titanium dioxide
ZnO	Zinc oxide
RO	Reverse Osmosis
FO	Forward Osmosis
ED	Electrodialysis
LIBs	Lithium-Ion Batteries
PEMFCs	Proton Exchange Membrane Fuel Cells
DMFCs	Direct Methanol Fuel Cells
VOCs	Volatile Organic Compounds
PCL/Gel	polycaprolactone/gelatin
EDC/NHS	1-Ethyl-3-(3-dimethylaminopropyl)-carbodiimide/N-hydroxysuccinimide
PVA	polyvinyl alcohol
CS	Chitosan
XG/GG/HA	xanthan gum/gellan gum/hyaluronan hydrogel
PAM	polyacrylamide
DN	Double network
DEA	N,N-diethylacrylamide
DMA	N,N-dimethylacrylamide
PVDF-HFP	poly(vinylidene fluoride-hexafluoro propylene
PBMA	poly (butyl methacrylate)
